# Correction: Oxa-376 and Oxa-530 variants of β-lactamase: computational study uncovers potential therapeutic targets of *Acinetobacter baumannii*

**DOI:** 10.1039/d2ra90089k

**Published:** 2022-09-12

**Authors:** Sajal Kumar Halder, Maria Mulla Mim, Md. Meharab Hassan Alif, Jannatul Fardous Shathi, Md. Nuhu Alam, Aparna Shil, Mahbubul Kabir Himel

**Affiliations:** Department of Biochemistry and Molecular Biology, Jahangirnagar University Savar Dhaka 1342 Bangladesh; Research Assistant at Padma Bioresearch Dhaka Bangladesh; Department of Pharmacy, Jahangirnagar University Savar Dhaka 1342 Bangladesh; Department of Botany, Jahangirnagar University Savar Dhaka 1342 Bangladesh himelbotju@juniv.edu aparna@juniv.edu

## Abstract

Correction for ‘Oxa-376 and Oxa-530 variants of β-lactamase: computational study uncovers potential therapeutic targets of *Acinetobacter baumannii*’ by Sajal Kumar Halder *et al.*, *RSC Adv.*, 2022, **12**, 24319–24338, https://doi.org/10.1039/D2RA02939A.

The authors regret that one of the authors' names, Md. Nuhu Alam, was spelt incorrectly in the original manuscript. The corrected list of authors for this paper is as shown above.

The Royal Society of Chemistry apologises for these errors and any consequent inconvenience to authors and readers.

## Supplementary Material

